# Human neuroinvasive Toscana virus infections in Italy from 2016 to 2023: Increased incidence in 2022 and 2023

**DOI:** 10.2807/1560-7917.ES.2025.30.2.2400203

**Published:** 2025-01-16

**Authors:** Emmanouil Alexandros Fotakis, Elisa Di Maggio, Martina Del Manso, Alberto Mateo-Urdiales, Daniele Petrone, Massimo Fabiani, Giulia Perego, Antonino Bella, Gioia Bongiorno, Ilaria Bernardini, Marco Di Luca, Giulietta Venturi, Claudia Fortuna, Stefania Giannitelli, Federica Ferraro, Francesco Maraglino, Patrizio Pezzotti, Anna Teresa Palamara, Flavia Riccardo, Italian Arbovirus Surveillance network, Italian Arbovirus Surveillance network, Daria Palmieri, Manuela Di Giacomo, Michele Labianca, Anna Domenica Mignuoli, Angelo D'Argenzio, Giovanna Mattei, Claudio Gualanduzzi, Matteo Giulio, Barbara Pellizzari, Schellenberger Cristina, Francesco Vairo, Camilla Sticchi, Lucia Crottogini, Danilo Cereda, Federica Attanasi, Sebastianelli Lolita, Fabio Filippetti, Manuela Mariano, Michele Colitti, Silvia Spertini, Zuccali Maria Grazia, Chiara Pasqualini, Daniela Lombardi, Rosa Prato, Martinelli Domenico, Maria Antonietta Palmas, Mario Palermo, Daniela Senatore, Francesco Angiò, Emanuela Balocchini, Foresi Simona, Arcelli Fabrizio, Sandra Ganio, Francesca Zanella, Debora Ballarin

**Affiliations:** 1 ECDC Fellowship Programme, Field Epidemiology path (EPIET), European Centre for Disease Prevention and Control (ECDC), Stockholm, Sweden; 2 Department of Infectious Diseases, Istituto Superiore Di Sanità, Rome, Italy; 3 Hygiene Unit, Department of Medical and Surgical Sciences, University of Foggia, Foggia, Italy; 4 Scuola di Specializzazione in Igiene e Medicina Preventiva, Università Vita-Salute San Raffaele, Milan, Italy; 5 Ministry of Health, Directorate-General for Health Prevention, Rome, Italy; 6 The members of the network are listed under Collaborators and at the end of the article

**Keywords:** Toscana virus, neuroinvasive infections, sand flies, Italy

## Abstract

**Background:**

Toscana virus (TOSV) is transmitted to humans through bites of infected sand flies. Neuroinvasive TOSV infections are leading causes of meningitis/encephalitis in southern Europe and notifiable in Italy since 2016. In 2022–23, Italy experienced extreme climate anomalies and a concomitant increase in mosquito and tick-borne disease transmission.

**Aim:**

To identify the spatiotemporal distribution and risk groups of neuroinvasive TOSV infections in Italy in 2022–23 vs 2016–21.

**Methods:**

We retrospectively described all autochthonous, laboratory-confirmed neuroinvasive TOSV cases notified to the national surveillance system in 2016–23 using frequencies, proportions, incidences and incidence risk ratios (IRRs) with 95% CIs, stratified by year, sex, age, region/autonomous province (AP) of infection/exposure and infection/exposure municipality by urbanisation level.

**Results:**

In 2022–23, 276 cases were notified (average annual incidence: 2.34/1,000,000 population) vs 331 cases in 2016–21 (0.92/1,000,000), with increased incidence extending into September. In 2022–23, infections were acquired in 12/21 regions/APs, predominantly in Emilia Romagna (57.6%; 159/276) as in 2016–21, including four regions/APs with no local infections in 2016–21. Similar to 2016–21, during 2022–23 residence in rural municipalities (vs urban), male sex, working age (19–67 years) and age > 67 years (vs ≤ 18 years) were identified as risk factors with IRRs of 2.89 (95% CI: 2.01–4.17), 2.17 (95% CI: 1.66–2.84), 5.31 (95% CI: 2.81–10.0) and 5.06 (95% CI: 2.59–9.86), respectively.

**Conclusion:**

Italy experienced a nearly 2.6-fold increase in neuroinvasive TOSV incidence in 2022–23 vs 2016–21. Raising public awareness on risk factors and personal protection measures may enhance prevention efforts.

**Key public health message**

**What did you want to address in this study and why?**

Toscana virus (TOSV), transmitted to humans by biting sandflies, is a major cause of central nervous system disorders in the Mediterranean region during summer. We wanted to examine whether the number of neuroinvasive TOSV infections increased in 2022–23 in Italy vs 2016–21, as observed for other vector-borne diseases carried by mosquitoes and ticks. We investigated the geographical distribution of infections and characteristics associated with increased infection risk.

**What have we learnt from this study?**

Compared with 2016–21, Italy experienced a 2.6-fold increase in the number of people with TOSV infections in 2022–23, with the peak of infections extending into autumn. Overall, most people over the 8-year time period were infected in north-central Italy, yet in increasing numbers in 2022–23. Male sex, rural area residence and age over 18 years were identified as risk factors for acquiring neuroinvasive TOSV infection.

**What are the implications of your findings for public health?**

It is important to inform the public, especially adults residing in rural areas, on sand fly exposure, the associated risk of TOSV neuroinvasive infection and on preventive measures reducing the risk of sand fly bites. Clinicians should also be aware of the increasing risk of TOSV infection during summer and early autumn months, and consider TOSV infection as possible diagnosis in patients presenting with clinical signs of meningitis or encephalitis.

## Introduction

Toscana virus (TOSV; *Toscana phlebovirus* species, *Phlebovirus* genus, *Phenuiviridae* family), first identified in 1971 in central Italy, is a negative-stranded RNA arbovirus transmitted to humans through bites of infected phlebotomine sand flies. As with several other arboviruses, TOSV is highly prevalent in Mediterranean countries [1] where, according to a study published in 2017, more than 250 million people are annually potentially exposed to the pathogen [2], mirroring the widespread distribution of competent vector species in the region [3]. Correlating with sand fly activity, human cases in European Mediterranean countries typically occur during the warm season, i.e. between June and October with a peak in August [3]. 

Tο date, the cycle of TOSV in nature remains elusive. Vertebrates serving as reservoirs have not yet been determined and humans are considered incidental – ‘dead end’ hosts, as infected individuals tend to exhibit transient and low level viraemia [4]. The majority of TOSV infections in humans are asymptomatic or mildly symptomatic, with an influenza-like illness [1,4]. However, TOSV demonstrates neurotropism and infections can lead to neuroinvasive manifestations following a median incubation period of 12 days [5]. Notably, neuroinvasive TOSV infections are among the leading causes of meningitis, encephalitis and meningo-encephalitis in southern European countries during the summer [4,6]. In addition, TOSV infections have also been associated with several peripheral neuropathies, e.g. Guillain–Barré syndrome [1]. 

Historic evidence from Italy corresponding to the late 1990s identifies TOSV as the aetiological agent of ca 40% of summer season meningitis cases [4]. Moreover, several studies conducted in the mid/late 2000s have found high TOSV seroprevalence rates ≥ 20% in central and southern Italy [7,8], concurrent with the strong presence of the primary viral vectors *Phlebotomus perniciosus* and *Phlebotomus perfiliewi* across the Italian territory [9,10]. Currently, there is no specific treatment or human vaccine available for neuroinvasive TOSV and diagnosis requires laboratory testing due to close resemblance of TOSV clinical manifestations with those of other neuroinvasive arboviral infections, e.g. West Nile virus (WNV), tick-borne encephalitis virus (TBEV)) [11]. Candidate tools available for TOSV prevention/response primarily involve vector control interventions and personal protection measures against sand fly bites.

Despite its public health significance, TOSV remains largely understudied and lacks national and international public health surveillance attention. Indicatively, neuroinvasive TOSV infections are not notifiable at the European Union/European Economic Area (EU/EEA) level and there is no standing EU case definition, although several case definition aspects are considered on the dedicated TOSV webpage of the European Centre for Disease Prevention and Control (ECDC) [12]. Moreover, several endemic EU/EEA countries report suboptimal TOSV molecular diagnostic capacity [13]. To the best of our knowledge, Italy is currently the only European country where neuroinvasive TOSV infections are notifiable at the national level, following the establishment of the national surveillance system in 2016. 

Existing literature suggests that the distribution and transmission intensity of arboviruses are largely driven and conditioned by several entomological parameters, e.g. vector distribution, abundance, and population dynamics, which are in turn heavily dependent on climatic conditions [14]. In 2022–23, Italy experienced climate anomalies, i.e. elevation of annual temperature, precipitation and relative humidity anomalies [15,16], and a concomitant increase in WNV, dengue virus and TBEV transmission [17], changes that possibly also apply to TOSV. To improve understanding of the spread of TOSV and its impact on human health supporting the planning of risk communication and risk management strategies, we aimed to describe the cases of neuroinvasive TOSV infection notified in Italy through the national surveillance system during the period 2016–23, by identifying their spatiotemporal distribution and population groups at risk for the years 2022–23 compared to 2016–21.

## Methods

### Surveillance system and data acquisition

Neuroinvasive TOSV infection is included in the mandatory notification system of the Italian Ministry of Health (MoH) and National Public Health Institute (ISS) since 2016. Physicians are obliged to report probable and confirmed cases to local/regional public health authorities (RPHAs), who then transfer the data to the MoH and ISS. All TOSV laboratory diagnoses are conducted in reference laboratories at the regional level. The national surveillance system assembles case-based information including demographics, clinical presentation, hospitalisation and laboratory test data, as well as data on place of most probable infection/exposure to distinguish imported from locally acquired infections, which also contains information on area of infection/exposure down to the municipality level for autochthonous cases. Surveillance is active all year round.

In November 2019, TOSV surveillance was integrated in the ‘National Plan for prevention, surveillance and response to Arboviruses 2020–2025’ developed by the MoH with the support of ISS and experts from RPHAs, which led to several surveillance system reformations [18]. Since 2020, cases are notified through a TOSV-specific surveillance form (vs a previous generic form), supported by a nationally uniform, composite case definition for neuroinvasive TOSV infections. Moreover, from 2020 onwards, case-based reporting from the RPHAs to MoH/ISS is conducted via an online platform (vs email/fax in the period 2016–19). From 2022, national level aggregated data reporting is systematically carried out through online dashboard bulletins published by ISS and updated on a weekly basis during the period of vector activity [17].

### Case definition, clinical characteristics and laboratory diagnostics

Italy recognises several clinical criteria and laboratory confirmatory tests in defining a confirmed case of neuroinvasive TOSV infection [18]. The clinical criteria are any person presenting fever, i.e. body temperature > 38 °C, and/or one of the following clinical manifestations: aseptic meningitis, meningo-encephalitis, encephalitis or polyneuropathy. The laboratory criteria comprise: (i) isolation of TOSV from cerebrospinal fluid (CSF) and/or other biological samples (blood, urine); (ii) identification of TOSV nucleic acid in CSF and/or other biological samples (blood, urine); (iii) identification of TOSV-specific IgM antibodies in CSF; (iv) identification of TOSV-specific IgM and IgG antibodies in serum; (v) seroconversion from a negative to positive titre, or four-fold increase in antibody titre for anti-TOSV antibodies in consecutive samples (at least 14 days apart). Confirmation of a case is possible when both the clinical and at least one of the listed laboratory criteria are met. Any person with the aforementioned clinical manifestations followed by laboratory detection of TOSV-specific IgM antibodies in serum is notified as a probable case. Cases are defined as autochthonous or imported based on their travel history and most probable area of exposure (in relation to the incubation period for TOSV and travel destination environment).

### Data sources and procedures

We obtained individual data from all TOSV laboratory-confirmed cases notified to the national surveillance system from May 2016 to November 2023, corresponding to eight transmission seasons. For the purposes of this study, only autochthonous cases that met the clinical criteria of a neuroinvasive infection were selected. Non-neuroinvasive infections were not considered, since they are probably more subject to under-ascertainment and, as they are not strictly under surveillance, are notified only sporadically. We extracted data from the two TOSV databases available at the national level: the first was compiled manually at ISS (relating to the period 2016–19) and the second corresponded to the online surveillance platform (relating to the period 2020–23). We aligned the variables common to both datasets to create a single database for the entire study period.

We retrieved census estimates of the Italian population from the Italian National Institute of Statistics (ISTAT) [19] stratified by year, sex (collected as a binary variable: male/female), region, municipality and age, which we categorised into three groups: 0–18, 19–67 (working age) and > 67 years (retirement age), for incidence calculation purposes. Data on the degree of urbanisation of the Italian municipalities for the years 2016–23 (categorised in three groups: urban, peri-urban and rural, by decreasing population density) were obtained from Eurostat [20] in order to calculate risk of infection by level of urbanisation. We used deterministic record linkage based on annual municipality identifiers to combine data from the three datasets.

### Statistical analysis

We performed a retrospective descriptive analysis on the notified neuroinvasive TOSV cases. Data corresponding to the period 2016–21, first analysed in a local publication [21], were subject to robust re-analysis for the purposes of this study. Data were analysed using frequencies, proportions and incidences per 1,000,000 population. Incidences were estimated for each notification year and the two notification periods (i.e. years 2016–21; 2022–23). Annual and period incidences were also stratified by sex, age, region/AP) of infection/exposure and infection/exposure municipality by urbanisation level. For notification period incidence estimates we calculated average annual incidences derived from the respective individual seasons within each period.

We carried out time series analysis techniques using negative binomial regression models to evaluate the overall trend and seasonality of notified cases by month of symptom onset. We adjusted our final models by date (progressive number of months from 2016 to 2023), annual seasonal components (estimated by sin (2πdate/12) and cos (2πdate/12)), and period of intense COVID-19 restriction measures (years 2020–21). In addition, we calculated crude incidence risk ratios (IRR) with 95% confidence intervals (CIs) to measure the associations between neuroinvasive TOSV infections and sex, age, and infection/exposure municipality by urbanisation level, using female sex, those ≤ 18 years of age and urban municipality residents as the respective reference groups. Incidence risk ratios were estimated using separate univariable negative binomial regression models for each notification period. We followed a similar approach to assess incidence differences between the two study periods, 2016–21 and 2022–23.

## Results

Over eight transmission seasons (2016–23), a total of 607 autochthonous neuroinvasive laboratory-confirmed TOSV cases were notified to the surveillance system of which 96.5% (580/601) were hospitalised, including two cases with a fatal outcome. Hospitalisation status was missing for six cases. Notified cases corresponded to an average annual incidence of 1.27 per 1,000,000 population (annual range: 0.58–2.59 per 1,000,000 population). The infection incidence by notification year, age group and sex is provided in Supplementary Table S1. Case median age was 47 years (age range: 0–91 years). Of the 607 cases, 69.2% (n = 420) were male and 30.8% (n = 187) were female. Regarding neurological syndromes, of all cases (n = 372) notified in the years 2020–23 (period during which detailed clinical data were available), 17.8% (n = 66) presented meningo-encephalitis, 32.5% (n = 121) encephalitis, 48.1% (n = 179) meningitis, 1.1% (n = 42) polyneuropathy and 0.5% (n = 2) meningitis and polyneuropathy.

### Seasonality and trend

**Figure 1:**
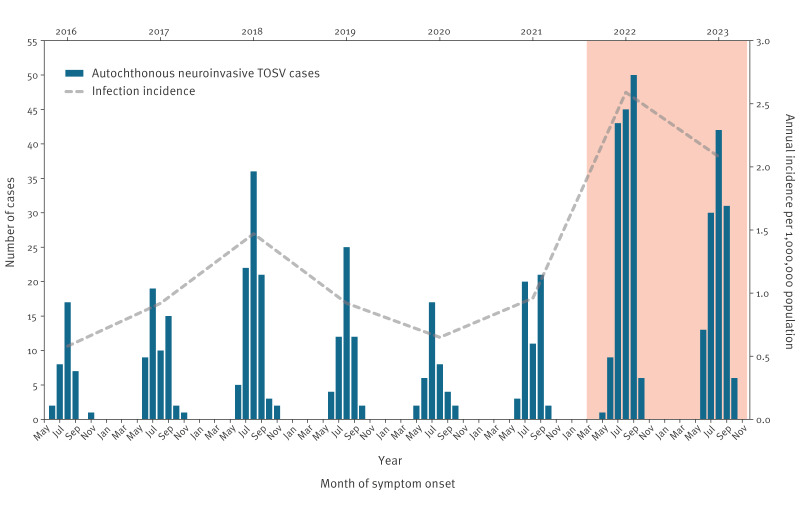
Confirmed autochthonous cases of neuroinvasive Toscana virus infection by month of symptom onset and annual incidence, Italy, 2016–2023 (n = 607 cases)

Overall, we found a statistically significant increasing trend of neuroinvasive TOSV infections over time (2016–23; IRR: 1.01; 95% CI: 1.01–1.02). The observed and predicted number of neuroinvasive TOSV cases by month of symptom onset is provided in Supplementary Figure S1. A total of 331 cases were notified in the period 2016–21, with an average annual incidence of 0.92 per 1,000,000 population. In this period, annual cases peaked in 2018 (n = 89 cases), dropping to 39 cases in 2020. In comparison, 276 cases (45.5% of 607 total cases) were notified in the period 2022–23 (average annual incidence of 2.34 per 1,000,000 population) (Figure 1). Risk of neuroinvasive infection was significantly higher in 2022–23 compared with 2016–2021 with an IRR of 2.55 (95% CI: 1.71–3.80).

TOSV: Toscana virus.

The dashed line corresponds to the annual autochthonous neuroinvasive infection incidence per 1,000,000 population and the shaded area represents the transmission seasons 2022 and 2023. Plotted data are up to December 2023.

We observed strong seasonality in neuroinvasive infections, extending between May–November, with clinical onset most prevalent in the period June–October (Figure 1). In both transmission periods (2016–21 and 2022–23) the majority of cases reported illness in August: 32.3% (107/331) and 31.5% (87/276) respectively. Compared with the years 2016–21, in 2022–23 case counts remained high throughout August (n = 87) and September (n = 81), with the proportion of cases reporting illness in September appearing to increase from 24% (80/331) in 2016–21 to 29.3% (81/276) in 2022–23 (Figure 1).

### Geographical distribution by region of infection and exposure

**Figure 2:**
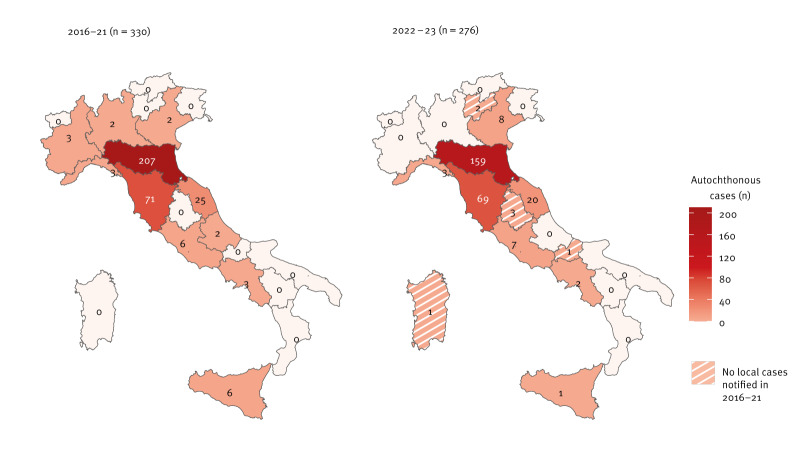
Geographical distribution of confirmed autochthonous cases of neuroinvasive Toscana virus infection, Italy, 2016–2023 (n = 606 cases)

In 2016–21, neuroinvasive TOSV infections were notified with exposure in 11 of 21 regions/APs (Figure 2). The majority of infection events (62.7%; 207/330) occurred in Emilia-Romagna with a regional average annual incidence of 7.75 per 1,000,000 population, followed by Tuscany (21.5%; 71/330) with an average annual incidence of 3.18 per 1,000,000 population. In 2022–23, cases occurred in 12 of 21 regions/APs. Similarly to the previous 6 years, the highest case counts were exposed/infected in Emilia-Romagna (57.6%; 159/276) and Tuscany (25.0%; 69/276). However, regional average annual incidences for this period appeared higher than in 2016–21, at 17.95 per 1,000,000 population for Emilia-Romagna and 9.42 per 1,000,000 population for Tuscany. Moreover, in this period cases acquired infections in the regions/APs of Sardinia (n = 1), Molise (n = 1), Umbria (n = 3) and Trento (n = 2), which had no known neuroinvasive infection occurrences in 2016–21 (Figure 2, see Supplementary Table S2 for the average annual incidence estimated for each region/AP). Conversely, in 2022–23, no cases were exposed in the regions of Abruzzo (n = 2 in 2016–21), Lombardy (n = 2 in 2016–21) and Piemonte (n = 3 in 2016–21).

Plotted numbers correspond to the cumulative number of cases by region/autonomous province (AP) of infection/exposure for each period (as notified in the national surveillance system). For 2016–21, a total of 330 cases are plotted rather than 331 as one case did not have an identified region of infection/exposure. Data are up to January 2024. A detailed map with the regions/APs of Italy is provided in Supplementary Figure S2.

The striped pattern signifies regions/APs where neuroinvasive infections were acquired for the first time in 2022 or 2023 according to notifications/case investigations for the period 2016–23.

### Case characteristics and risk factors for neuroinvasive TOSV infection

In both 2016–21 and 2022–23, the majority of cases were male (respective relative frequencies: 70.7% and 67.4%), between 19 and 67 years of age (74.9% and 73.9%) and exposed/infected in peri-urban municipalities (42.0% and 48.9%) (Table). Throughout our study period, the population groups with highest risk of infection were males (cf.d with females), with IRRs of 2.55 (95% CI: 1.75–3.71) in 2021–16 and 2.17 (95% CI: 1.66–2.84) in 2022–23 and those aged from 19 to 67 years of age (cf.d with those < 18 years), with IRRs of 2.89 (95% CI: 1.73–4.82) and 5.31 (95% CI: 2.81–10.00) in 2021–16 and 2022–23, respectively (Table). Upon sex and age stratification, the highest risk was estimated for males over 67 years of age in the period 2022–23, with an average annual incidence of 4.87 per 1,000,000 population. 

**Table:** Autochthonous neuroinvasive Toscana virus infection case characteristics and demographic risk factors, Italy, 2016–2023 (n = 607 cases)

Variables	2016–21 (n = 331)					2022–23 (n = 276)				
	Cases		Incidence per million^a^	IRR	95% CI	Cases		Incidence per million^a^	IRR	95% CI
	n	%				n	%			
**Age**										
0–18	23	6.9	0.37	Reference		10	3.6	0.52	Reference	
19–67	248	74.9	1.08	2.89	1.73–4.82	204	73.9	2.73	5.31	2.81–10.0
> 67	60	18.1	0.86	2.31	1.33–4.04	62	22.5	2.60	5.06	2.59–9.86
**Sex**										
Female	97	29.3	0.52	Reference		90	32.6	1.48	Reference	
Male	234	70.7	1.33	2.55	1.75–3.71	186	67.4	3.23	2.17	1.66–2.84
**Urbanicity^b^**										
Urban	82	25.3	0.64	Reference		57	21.3	1.36	Reference	
Peri-urban	136	42.0	0.79	1.23	0.81–1.86	131	48.9	2.32	1.70	1.21–2.38
Rural	106	32.7	1.71	2.65	1.74–4.05	80	29.9	3.96	2.89	2.01–4.17

CI: confidence interval; IRR: Incidence risk ratio.

^a^ Average annual incidence for each period.

^b^ Total number of cases with known municipality of exposure: 2016–21 (n = 324); 2022–23 (n = 268).

Finally, the risk of contracting an infection was highest in rural municipalities (cf.d with urban municipalities); IRRs of 2.65 (95% CI: 1.74–4.05) in 2016–21 and 2.89 (95% CI: 2.01–4.17) in 2022–23. Risk of infection increased significantly across all municipality types (by urbanisation level) in 2022–23 compared with 2016–21 with IRRs of 2.32 (95% CI: 1.51–3.54) in rural areas, 2.93 (95% CI: 1.86–4.62) in peri-urban areas and 2.12 (95% CI: 1.40–3.20) in urban areas.

## Discussion

Italy experienced an increasing trend of autochthonous neuroinvasive TOSV infections over the years 2016–23. Specifically, the 2022–23 period was characterised by a heightened incidence pattern extending into autumn, incidence increases in historically highly impacted regions, and reported infection exposure in regions where TOSV infections had not been previously acquired according to notifications/case investigations in the years 2016–21. Male sex, working and retirement age and residence in rural municipalities were identified as risk factors for neuroinvasive TOSV infection.

To date, national scale evidence on the burden and trend of neuroinvasive TOSV infections in other EU/EEA countries remains scarce. Nonetheless, human seroprevalence studies conducted in Spain, Portugal, France and several Balkan countries (e.g. Greece, Bulgaria) between 2010 and 2020, indicate significant levels of autochthonous transmission, with healthy population TOSV seroprevalence rates often exceeding 20% [1,3].

The decision of including neuroinvasive TOSV infections under systematic surveillance in Italy has indeed afforded case detection and notification leading to an increase in observations that is important to better understanding the epidemiology and prevalent characteristics of the affected populations, although room for improvement exists. Expanding the surveillance of TOSV in other EU countries could advance our understanding of its spread and burden in Europe.

Our finding of significantly higher neuroinvasive laboratory-confirmed TOSV case counts in the years 2022–23 compared with 2016–21 could in part reflect a potential increase in TOSV testing attributed to the surveillance system improvements implemented as of 2020. Nonetheless, the TOSV infection trend we observed, including all annual case count fluctuations, closely mirrors the autochthonous incidence trends of TBE, WNV (i.e. arboviral infections reported as per the EU case definition throughout the study period) and leishmaniasis. Specifically, all aforementioned pathogens, which are subject to distinct surveillance programmes, exhibited heightened transmission seasons in 2022–23 [17,22].

Additionally, previous evidence from Emilia-Romagna [23], the region reporting the majority of cases in both study periods, indicates systematic laboratory ascertainment that pre-dates the surveillance system reform in 2019–20, providing further evidence of an increasing TOSV trend over our study period. Overall, the combination of the aforementioned factors indicates a significant increase in the incidence of severe TOSV infections in 2022–23, regardless of any potential changes in the surveillance system sensitivity levels.

Notably, the transmission seasons with highest case counts (i.e. 2018, 2022, 2023), including patterns of heightened infection occurrence in early autumn for the years 2022–23, align with concurrent striking temperature anomalies in the same years. Specifically, the 2018 spring/summer season temperature in several northern regions in Italy was higher compared with that of the immediately preceding and succeeding years by a minimum of + 0.66° C, i.e. average weekly median temperature anomaly [16]. Moreover, 2022 was the warmest year on record since 1961 with a positive mean temperature anomaly of + 1.12 °C compared with the period 1991–2020 [15], while the summer of 2023 was warmer compared with the corresponding 1991–2020 climatological averages [16].

The dominant TOSV vectors in Italy, *P. perniciosus* and *P. perfiliewi* require warm temperatures for their development and survival across all life stages [3]. Considering the species' thermophilic nature, it is likely that the recorded temperature patterns in 2018, and 2022–23, prompted earlier, intensified, temporally prolonged and geographically extended sand fly activity in the corresponding years [10,24], conditioning elevated TOSV transmission levels.

Precipitation anomalies may have also influenced the observed incidence patterns. TOSV incidence peaked in 2022, which was recorded as the driest year in Italy since 1961, with an especially strong negative precipitation anomaly recorded in spring [15]. Several studies from Italy show a negative association between precipitation and sand fly abundance, especially during the spring/summer season [10,25], likely explained by heavy rainfall increasing larval death and reducing adult flight activity and resting site availability [26]. Reverse mechanisms applying amidst the drier 2022 spring season may have facilitated increased sand fly survival and activity, ultimately elevating TOSV transmission.

Conversely to 2018 and 2022–23, case numbers were relatively low in 2020 and 2021. These years coincide with the acute phase of the COVID-19 pandemic in Italy, when public health and social measures (PHSMs) were implemented throughout the country. The PHSMs were most stringent in 2020, during which regional and national lockdowns were imposed, while restriction measures gradually relaxed in the spring of 2021. Inevitably, PHSMs reduced human mobility and subsequently most likely the general risk of exposure to sand fly bites. Moreover, evidence from Italy shows that the pandemic altered peoples’ overall healthcare seeking behaviour, likely temporarily increasing neuroinvasive TOSV under-ascertainment [27].

Throughout 2016–23, we observed higher neuroinvasive TOSV case counts in Emilia-Romagna and Tuscany compared with all other regions. Vector environmental suitability may in part explain this geographical pattern, as demonstrated by the high abundance of *P. perniciosus* and *P. perfiliewi* in several sub-regional foci, especially in the northern foothills in Emilia-Romagna [28]. However, explanatory factors more likely pertain to non-uniform diagnostic assessment protocols applied across the country, e.g. in Emilia-Romagna all patients testing negative for WNV and Usutu virus are systematically tested for TOSV, as well as inter-regional healthcare service variations influencing infection ascertainment at the community-level. The latter factors could in part also underly the finding of infection occurrences in the regions/APs of Sardinia, Molise, Umbria and Trento in the period 2022–23 vs 2016–21, especially upon considering pre-2016 seroprevalence findings from the respective regions [29,30]. 

Neuroinvasive TOSV incidence was highest in rural municipalities, consistent with TOSV vector ecology whereby sand fly larvae typically develop in environments rich in decomposing organic material, at a relatively constant temperature and in almost total darkness, i.e. environments most often found in contexts with a low level of urbanisation [31]. Nonetheless, in line with a recent study from Spain [32], infections were also acquired in peri-urban and urban settings, likely reflecting vector ecological plasticity traits enabling adaptation to human-made environments. In particular, the significant increase in the incidence of neuroinvasive infections in urban and peri-urban municipalities in 2022–23 compared with previous years needs to be monitored, as it indicates the potential for a more elevated TOSV transmission in ecological settings where human density is higher [33].

In line with previous studies [7,8], male sex and working age were identified as risk factors for neuroinvasive TOSV infection. The higher infection incidences among these groups probably reflect an increased exposure risk to sand fly bites. Specifically, outdoor manual jobs, especially in rural and peri-urban settings, are traditionally undertaken by men, while working age individuals likely spend longer times outdoors in high-risk transmission settings amid their occupational activities, compared with younger individuals. We also observed an increased risk of infection in the older population over 67 years of age. Older age is likely accompanied by a higher risk of TOSV-associated neurological complications [34], as shown for other arboviruses such as WNV.

Our study has several limitations. Firstly, our results may underestimate the true number of neuroinvasive TOSV infections and portray a somewhat biased geographical distribution because of the diverse impact of under-diagnosis and under-ascertainment, varying by administrative region. Secondly, as our study focused on neuroinvasive TOSV infections, our findings underestimate the true incidence and distribution of TOSV in Italy, especially when considering that a significant proportion of TOSV infections are non-neuroinvasive [4,11]; indicatively between 2020 and 2023, 79 TOSV cases lacking neuro-invasive manifestations were notified to the national surveillance system. Thirdly, in the absence of data regarding the number of TOSV diagnostic analyses conducted in 2022–23 vs previous years, we were unable to quantitatively assess the effect of the surveillance system revisions on the increase of TOSV notifications. Fourthly, when calculating incidence by exposure municipality urbanisation level, we used the corresponding municipalities' resident population size as a denominator. However, this does not take into account population movements between municipalities of different urbanisation status, potentially resulting in a slight over or under estimation of the true IRRs. Finally, as the surveillance system lacks mid- and long-term sequelae information on notified neuroinvasive cases, hospitalisation and death outcomes aside we were not able to capture the true impact of TOSV on individual health.

## Conclusions

Italy experienced a substantial increase in TOSV incidence in 2022–23 compared with previous years. Similar incidence patterns may have also occurred in neighbouring countries with a documented TOSV transmission history and extended vector distribution. Our findings highlight the need of raising public awareness in Italy to prevent sand fly exposure by adopting risk-mitigating behaviours, with a special focus on those of working age, men over 67 years of age and individuals residing in rural municipalities. In addition, raising awareness among clinicians of the potential expansion of high transmission temporality may further encourage considering TOSV in the differential diagnosis of patients presenting neuroinvasive infection symptomatology during late summer and autumn. Ultimately, establishing national level integrated human and entomological TOSV surveillance activities may greatly facilitate early TOSV detection, targeted interventions and evidence-based resource allocation, counteracting the increasing trend of neuroinvasive infections.

## Italian Arbovirus Surveillance network

Daria Palmieri, Manuela Di Giacomo (Abruzzo); Michele Labianca (Basilicata); Anna Domenica Mignuoli (Calabria); Angelo D'Argenzio (Campania); Giovanna Mattei, Claudio Gualanduzzi, Matteo Giulio (Emilia-Romagna); Barbara Pellizzari, Schellenberger Cristina (Friuli Venezia Giulia); Francesco Vairo (Lazio); Camilla Sticchi (Liguria); Lucia Crottogini, Danilo Cereda, Federica Attanasi (Lombardia); Sebastianelli Lolita, Fabio Filippetti (Marche); Manuela Mariano, Michele Colitti (Molise); Silvia Spertini (Pa Bolzano); Zuccali Maria Grazia (Pa Trento); Chiara Pasqualini, Daniela Lombardi (Piemonte); Rosa Prato, Martinelli Domenico (Puglia); Maria Antonietta Palmas (Sardegna); Mario Palermo (Sicilia); Daniela Senatore, Francesco Angiò, Emanuela Balocchini (Toscana); Foresi Simona, Arcelli Fabrizio (Umbria); Sandra Ganio (Valle D’Aosta); Francesca Zanella, Debora Ballarin (Veneto).

## Supplementary Data

249000 Supplement

## References

[1] Ayhan N Charrel RN . An update on Toscana virus distribution, genetics, medical and diagnostic aspects. Clin Microbiol Infect. 2020;26(8):1017-23. 10.1016/j.cmi.2019.12.015 31904562

[2] Ayhan N Baklouti A Prudhomme J Walder G Amaro F Alten B Practical guidelines for studies on sandfly-borne phleboviruses: part i: important points to consider ante field work. Vector Borne Zoonotic Dis. 2017;17(1):73-80. 10.1089/vbz.2016.1957 28055576

[3] Ayhan N Prudhomme J Laroche L Bañuls AL Charrel RN . Broader geographical distribution of Toscana virus in the Mediterranean region suggests the existence of larger varieties of sand fly vectors. Microorganisms. 2020;8(1):114. 10.3390/microorganisms8010114 31947561 PMC7022675

[4] Charrel RN Gallian P Navarro-Marí JM Nicoletti L Papa A Sánchez-Seco MP Emergence of Toscana virus in Europe. Emerg Infect Dis. 2005;11(11):1657-63. 10.3201/eid1111.050869 16318715 PMC3367371

[5] Laroche L Jourdain F Ayhan N Bañuls AL Charrel R Prudhomme J . Incubation period for neuroinvasive Toscana virus infections. Emerg Infect Dis. 2021;27(12):3147-50. 10.3201/eid2712.203172 34808074 PMC8632186

[6] Depaquit J Grandadam M Fouque F Andry PE Peyrefitte C . Arthropod-borne viruses transmitted by Phlebotomine sandflies in Europe: a review. Euro Surveill. 2010;15(10):19507. 10.2807/ese.15.10.19507-en 20403307

[7] Terrosi C Olivieri R Bianco C Cellesi C Cusi MG . Age-dependent seroprevalence of Toscana virus in central Italy and correlation with the clinical profile. Clin Vaccine Immunol. 2009;16(8):1251-2. 10.1128/CVI.00376-08 19553552 PMC2725527

[8] Calamusa G Valenti RM Vitale F Mammina C Romano N Goedert JJ Seroprevalence of and risk factors for Toscana and Sicilian virus infection in a sample population of Sicily (Italy). J Infect. 2012;64(2):212-7. 10.1016/j.jinf.2011.11.012 22120113 PMC3630500

[9] Defilippo F Carrera M Lelli D Canziani S Moreno A Sozzi E Distribution of phlebotomine sand flies (Diptera: Psychodidae) in the Lombardy Region, Northern Italy. Insects. 2022;13(5):463. 10.3390/insects13050463 35621798 PMC9146192

[10] Calzolari M Romeo G Munari M Bonilauri P Taddei R Sampieri M Sand Flies and Pathogens in the Lowlands of Emilia-Romagna (Northern Italy). Viruses. 2022;14(10):2209. 10.3390/v14102209 36298764 PMC9608450

[11] Charrel RN Bichaud L de Lamballerie X . Emergence of Toscana virus in the mediterranean area. World J Virol. 2012;1(5):135-41. 10.5501/wjv.v1.i5.135 24175218 PMC3782275

[12] European Centre for Disease Prevention and Control (ECDC). Toscana virus infection. Stockholm: ECDC. [Accessed: 10 Dec 2024]. Available from: https://www.ecdc.europa.eu/en/toscana-virus-infection

[13] Reusken C Baronti C Mögling R Papa A Leitmeyer K Charrel RN . Toscana, West Nile, Usutu and tick-borne encephalitis viruses: external quality assessment for molecular detection of emerging neurotropic viruses in Europe, 2017. Euro Surveill. 2019;24(50):1900051. 10.2807/1560-7917.ES.2019.24.50.1900051 31847946 PMC6918591

[14] Paz S . Climate change impacts on vector-borne diseases in Europe: Risks, predictions and actions. Lancet Reg Health Eur. 2020;1:100017. 10.1016/j.lanepe.2020.100017 35104838 PMC8454730

[15] Italian Institute for Environmental, Protection and Research. The Italian climate in 2022: a preliminary assessment. 2022. Available from: https://scia.isprambiente.it

[16] Copernicus. Surface air temperature maps. Paris: European Space Agency (ESA). [Accessed: 14 Feb 2024]. Available from: https://climate.copernicus.eu/surface-air-temperature-maps

[17] Istituto Superiore di Sanità. Arbovirosi in Italia 2024. [Arboviruses in Italy 2024]. Rome: Istituto Superiore di Sanità (ISS). [Accessed: 12 Feb 2024]. Available from: https://www.epicentro.iss.it/arbovirosi/dashboard

[18] Italian Ministry of Health. Piano Nazionale di prevenzione, sorveglianza e risposta alle Arbovirosi (PNA) 2020-2025. [National Plan for prevention, surveillance and response to Arboviruses 2020-2025]. Rome: Italian Ministry of Health; 2019. Italian. Available from: https://www.salute.gov.it/imgs/C_17_pubblicazioni_2947_allegato.pdf

[19] Italian National Institute of Statistics. Population and Households. [Accessed: 2 Feb 2024]. Available from: https://www.istat.it/en/population-and-households?data-and-indicators

[20] Eurostat. Local administrative units. Luxembourg: Eurostat. [Accessed: 5 Nov 2023]. Available from: https://ec.europa.eu/eurostat/web/nuts/local-administrative-units

[21] Mellace F Del Manso M Oradini-Alacreu A Ceccarelli E Mateo-Urdiales A Petrone D Meningiti, meningo-encefaliti ed encefaliti da virus Toscana in Italia, 2016-2021: punta dell’iceberg di una arbovirosi endemica poco conosciuta. [Meningitis, meningoencephalitis and encephalitis from Toscana virus in Italy, 2016-2021: the tip of the iceberg of a little-known endemic arbovirus]. Boll Epidemiol Naz. 2022;3(2):10-9. Italian.

[22] Todeschini R Musti MA Pandolfi P Troncatti M Baldini M Resi D Re-emergence of human leishmaniasis in northern Italy, 2004 to 2022: a retrospective analysis. Euro Surveill. 2024;29(4):2300190. 10.2807/1560-7917.ES.2024.29.4.2300190 38275016 PMC10986649

[23] Azzalini D, Belloli GL, Gualanduzzi C, Mattei G, Matteo G, Mattivi A, et al. Toscana virus in Emilia-Romagna. Bologna: Settore Prevenzione collettiva e Sanità pubblica - Regione Emilia-Romagna; 2022. Italian. Available from: https://www.epicentro.iss.it/territorio/emilia-romagna/pdf/Epidemiologia-Toscana-Virus-2022.pdf

[24] Alten B Maia C Afonso MO Campino L Jiménez M González E Seasonal dynamics of phlebotomine sand fly species proven vectors of mediterranean leishmaniasis caused by Leishmania infantum. PLoS Negl Trop Dis. 2016;10(2):e0004458. 10.1371/journal.pntd.0004458 26900688 PMC4762948

[25] Moirano G Ellena M Mercogliano P Richiardi L Maule M . Spatio-temporal pattern and meteo-climatic determinants of visceral leishmaniasis in Italy. Trop Med Infect Dis. 2022;7(11):337. 10.3390/tropicalmed7110337 36355879 PMC9694427

[26] Simsek FM Alten B Caglar SS Ozbel Y Aytekin AM Kaynas S Distribution and altitudinal structuring of phlebotomine sand flies (Diptera: Psychodidae) in southern Anatolia, Turkey: their relation to human cutaneous leishmaniasis. J Vector Ecol. 2007;32(2):269-79. 10.3376/1081-1710(2007)32[269:DAASOP]2.0.CO;2 18260517

[27] Gualano MR Corradi A Voglino G Bert F Siliquini R . Beyond COVID-19: a cross-sectional study in Italy exploring the covid collateral impacts on healthcare services. Health Policy. 2021;125(7):869-76. 10.1016/j.healthpol.2021.03.005 33840478 PMC7987502

[28] Calzolari M Romeo G Callegari E Bonilauri P Chiapponi C Carra E Co-circulation of phleboviruses and leishmania parasites in sand flies from a single site in Italy monitored between 2017 and 2020. Viruses. 2021;13(8):1660. 10.3390/v13081660 34452524 PMC8402820

[29] Francisci D Papili R Camanni G Morosi S Ferracchiato N Valente M Evidence of Toscana virus circulation in Umbria: first report. Eur J Epidemiol. 2003;18(5):457-9. 10.1023/A:1024295710118 12889693

[30] Venturi G Madeddu G Rezza G Ciccozzi M Pettinato ML Cilliano M Detection of Toscana virus central nervous system infections in Sardinia Island, Italy. J Clin Virol. 2007;40(1):90-1. 10.1016/j.jcv.2007.06.005 17662649

[31] Feliciangeli MD . Natural breeding places of phlebotomine sandflies. Med Vet Entomol. 2004;18(1):71-80. 10.1111/j.0269-283X.2004.0487.x 15009450

[32] Gámbaro F Pérez AB Prot M Agüera E Baidaliuk A Sánchez-Seco MP Untargeted metagenomic sequencing identifies Toscana virus in patients with idiopathic meningitis, southern Spain, 2015 to 2019. Euro Surveill. 2023;28(45):2200913. 10.2807/1560-7917.ES.2023.28.45.2200913 37943504 PMC10636744

[33] Fournet F Simard F Fontenille D . Green cities and vector-borne diseases: emerging concerns and opportunities. Euro Surveill. 2024;29(10):2300548. 10.2807/1560-7917.ES.2024.29.10.2300548 38456216 PMC10986671

[34] Christova I Panayotova E Trifonova I Taseva E Gladnishka T Ivanova V . Serologic evidence of widespread Toscana virus infection in Bulgaria. J Infect Public Health. 2020;13(2):164-6. 10.1016/j.jiph.2019.07.008 31401037

