# Supplementary material for: Human neuroinvasive Toscana virus infections in Italy from 2016 to 2023: Increased incidence in 2022 and 2023
Source: Euro Surveill. 2025 Jan 16;30(2):2400203. doi: 10.2807/1560-7917.ES.2025.30.2.2400203 (PMC11740290; doi:10.2807/1560-7917.ES.2025.30.2.2400203)
Supplement: Supplement [file 24-00203_FOTAKIS_Supplement.pdf]

Supplementary file: TOSV trend/seasonality and incidence per 1,000,000 population by case characteristics

This supplementary material is hosted by Eurosurveillance as supporting information alongside the article [Human neuroinvasive Toscana virus infections in Italy from 2016 to 2023: Increased incidence in 2022 and 2023] on behalf of the authors who remain responsible for the accuracy and appropriateness of the content. The same standards for ethics, copyright, attributions and permissions as for the article apply. Eurosurveillance is not responsible for the maintenance of any links or email addresses provided therein.

Supplementary Table 1: Autochthonous neuroinvasive *Toscana* virus infection incidence per 1,000,000 population by year/period, age group and sex, 2016-2023, Italy (n = 607 cases).

Characteristics		Year - Period									
Age group	Sex	2016	2017	2018	2019	2020	2021	2022	2023	2016-2021*	2022-2023*
0-18	Female	0.19	0.59	0.20	0.41	0.21	0.21	0.21	0.00	0.30	0.10
0-18	Male	0.00	0.56	0.75	0.57	0.39	0.39	0.80	1.00	0.44	0.90
0-18	Both	0.09	0.57	0.48	0.49	0.30	0.30	0.51	0.52	0.37	0.52
19-67	Female	0.46	0.36	1.19	0.63	0.32	0.58	2.08	2.03	0.59	2.05
19-67	Male	0.93	1.82	2.66	1.37	1.17	1.50	3.87	2.95	1.57	3.41
19-67	Both	0.69	1.08	1.92	1.00	0.74	1.04	2.97	2.49	1.08	2.73
>67	Female	0.62	0.61	0.45	0.45	0.30	0.59	1.04	0.74	0.50	0.89
>67	Male	0.64	0.83	1.42	1.81	1.19	2.17	5.87	3.87	1.34	4.87
>67	Both	0.63	0.70	0.87	1.03	0.68	1.27	3.12	2.09	0.86	2.60
All	Female	0.45	0.45	0.87	0.55	0.29	0.53	1.55	1.42	0.52	1.48
All	Male	0.71	1.43	2.11	1.30	1.03	1.42	3.69	2.78	1.33	3.23
All	Both	0.58	0.92	1.47	0.92	0.65	0.96	2.59	2.08	0.92	2.34

*Average annual incidence

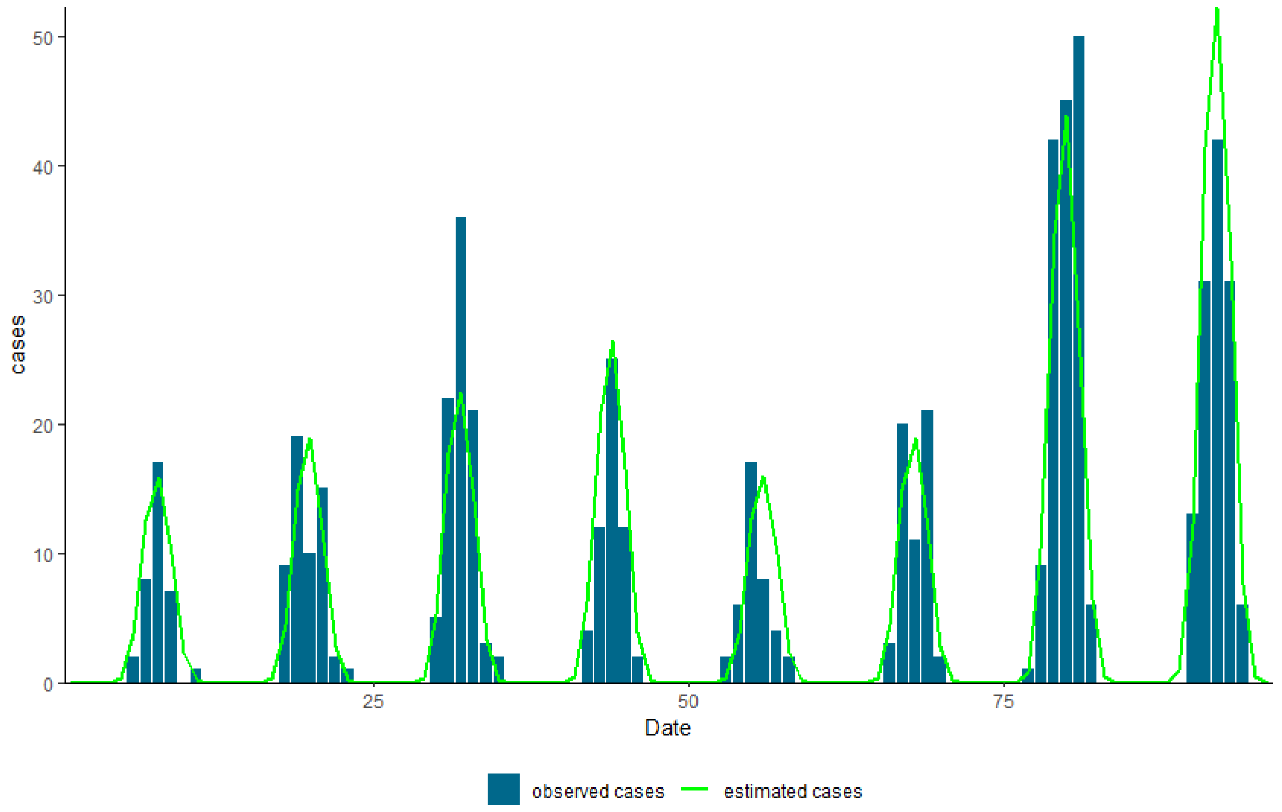

Supplementary Figure 1: Observed and predicted number of neuroinvasive *Toscana* virus cases by month of symptom onset; adjusted by date (progressive number of months from 2016 to 2023), annual seasonal components (estimated by sin (2πdate/12) and cos (2πdate/12)), and period of intense COVID-19 restriction measures (2020-2021), Italy, 2016-2023 (n = 607 cases). Data for 2023 are under consolidation.

Supplementary Table 2: Cases and average annual incidence per 1,000,000 population of autochthonous neuroinvasive *Toscana* virus infection by period and region/autonomous province of exposure/infection, 2016-2023, Italy (n = 607 cases).

Period	Region/Autonomous Province	cases (n)	Average annual incidence per 1,000,000 population
2016-2021*	Abruzzo	2	0.25
	Campania	3	0.08
	Emilia-Romagna	207	7.75
	Lazio	6	0.17
	Liguria	3	0.32
	Lombardia	2	0.03
	Marche	25	2.71
	Piemonte	3	0.11
	Sicilia	6	0.20
	Toscana	71	3.18
	Veneto	2	0.07
2022-2023^	Campania	2	0.18
	Emilia-Romagna	159	17.95
	Lazio	7	0.61
	Liguria	3	0.99
	Marche	20	6.73
	Molise	1	1.72
	Sardegna	1	0.32
	Sicilia	1	0.10
	Toscana	69	9.42
	Trento	2	1.84
	Umbria	3	1.75
	Veneto	8	0.82

*A total of 330 cases are described as 1 case did not have an identified region of infection/exposure

^ Data for 2023 are under consolidation

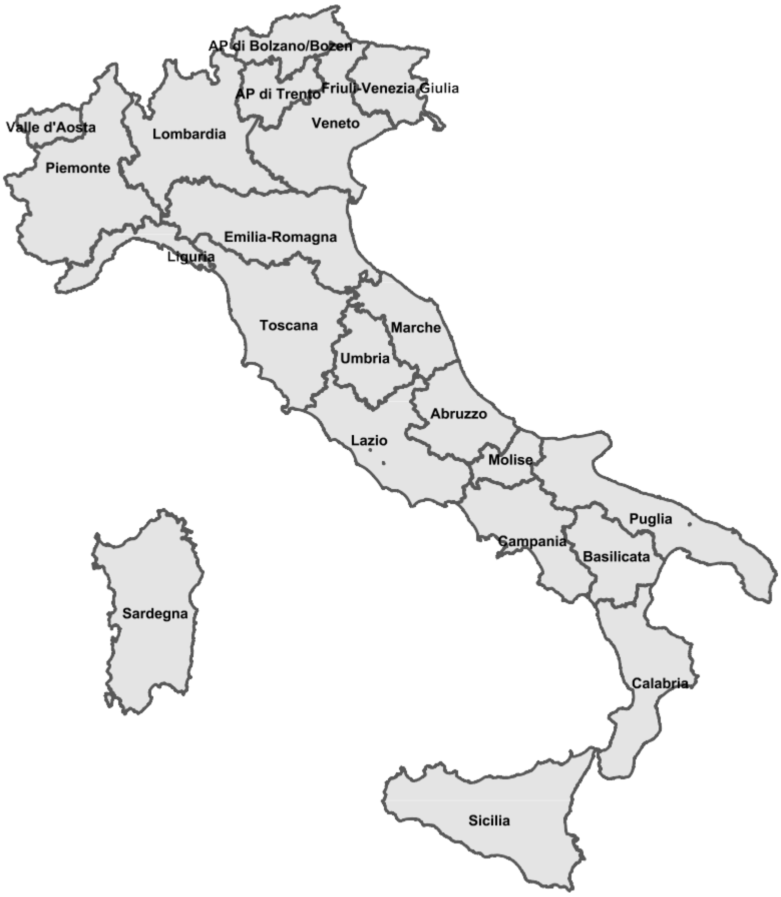

Supplementary Figure 2: Map of Italy, with all Autonomous Provinces and Regions, 2023.
